# Xiaohua Funing decoction ameliorates non-alcoholic fatty liver disease by modulating the gut microbiota and bile acids

**DOI:** 10.3389/fmicb.2025.1511885

**Published:** 2025-02-10

**Authors:** Yan Li, Jindong Zhao

**Affiliations:** ^1^Department of Infectious Diseases, The First Affiliated Hospital of Anhui University of Chinese Medicine, Hefei, Anhui, China; ^2^Department of Endocrinology, The First Affiliated Hospital of Anhui University of Chinese Medicine, Hefei, China; ^3^Center for Xin’an Medicine and Modernization of Traditional Chinese Medicine of IHM, The First Affiliated Hospital of Anhui University of Chinese Medicine, Hefei, Anhui, China

**Keywords:** non-alcoholic fatty liver disease, Xiaohua Funing decoction, gut microbiota, bile acids, *Bacteroidales_bacterium*, glycochenodeoxycholic acid

## Abstract

**Introduction:**

The gut microbiota and bile acids (BAs) have emerged as factors involved in the development of non-alcoholic fatty liver disease (NAFLD). Xiaohua Funing decoction (XFD) is a traditional Chinese medicine formula used for the treatment of NAFLD. Previous studies have indicated that XFD protects liver function, but the underlying mechanism remains unclear.

**Methods:**

In this study, a Wistar rat model of NAFLD (Mod) was established via a high-fat diet. The effects of obeticholic acid (OCA) and XFD on Mod rats were subsequently evaluated. Wistar rats in the control (Con) group were fed a standard diet. There were eight rats in each group, and the treatment lasted for 12 weeks. Furthermore, metagenomic sequencing and BA metabolomic analyses were performed.

**Results:**

Compared to the Con group, the Mod group presented significant differences in body and liver weights; serum total cholesterol (TC) and triglyceride (TG) levels; and liver TG, TC, and bile salt hydrolase levels (*p* < 0.05 or *p* < 0.01). Importantly, OCA and XFD administration normalized these indicators (*p* < 0.05 or *p* < 0.01). Pathology of the liver and white fat steatosis was observed in the Mod group, but steatosis was significantly alleviated in the OCA and XFD groups (*p* < 0.05 or *p* < 0.01). The abundances of *Bacteroidales_bacterium*, *Prevotella_*sp., *bacterium_0.1xD8-71*, and *unclassified_g_Turicibacter* in the Mod group were significantly different from those in the Con group (*p* < 0.05 or *p* < 0.01), whereas the abundance of *Bacteroidales_bacterium* was greater in the XFD group. A total of 17, 24, and 24 differentially abundant BAs were detected in the feces, liver, and serum samples from the Mod and Con groups, respectively (*p* < 0.05 or *p* < 0.01). In the feces, liver, and serum, XFD normalized the levels of 16, 23, and 14 BAs, respectively, including glycochenodeoxycholic acid, deoxycholic acid, murideoxycholic acid, lithocholic acid, 23-nordeoxycholic acid, and 3β-ursodeoxycholic acid. In addition, glycochenodeoxycholic acid was identified as a potential biomarker of NAFLD.

**Discussion:**

In summary, our experiments revealed that XFD regulates the gut microbiota and BAs, providing beneficial effects on liver lipid accumulation in NAFLD.

## Introduction

Non-alcoholic fatty liver disease (NAFLD) is a common chronic liver disease with an incidence rate of approximately 30% (Rinella, 2015) and has become an increasingly severe public health concern. In particular, NAFLD may progress to cirrhosis and hepatocellular carcinoma (Younossi, 2019). Studies have shown that the pathogenesis of fatty liver disease is related to genetic susceptibility, environmental factors, metabolic disorders, autophagy, the gut microbiota, and metabolites (Song et al., 2015; Bauer et al., 2022; Aron-Wisnewsky et al., 2020). Although a causative claim about the gut microbiota in NAFLD is challenging, some meaningful exploratory research is being conducted (Vallianou et al., 2021). Diet interventions, probiotics, and fecal microbiota transfer can modify the gut microbiota to alleviate NAFLD (Tilg et al., 2021). The gut microbiota is involved in the metabolism of bile acids (BAs). BAs are molecules that regulate NAFLD (Chávez-Talavera et al., 2017). The farnesoid X receptor (FXR), a BA receptor, may be a potential target for NAFLD treatment (Tian et al., 2022; Zhou et al., 2020). Obeticholic acid (OCA), a BA FXR agonist, has been developed as a candidate medication for NAFLD therapy (Wang et al., 2024; Ahmed et al., 2022). Thus, we chose OCA as a positive control drug. The gut microbiota structure and composition of BAs, especially *Blautia* and taurine-bound BAs, changed after treatment with OCA (Zhang et al., 2019).

NAFLD belongs to the Gan pi category in traditional Chinese medicine (TCM). In recent decades, studies have confirmed that TCM has the potential to treat NAFLD. These treatments include Dachaihu decoction, Lingguizhugan decoction, Shenling Baizhu powder, and berberine (Yang et al., 2019; Zhu et al., 2023; Zhang et al., 2018; Wu et al., 2019). We speculate that the pathogenesis of NAFLD is related to liver depression and spleen deficiency. Xiaohua Funing decoction (XFD) is a TCM prescription. In the present study, we demonstrated that XFD has treatment benefits for NAFLD as it can regulate lipid levels and protect liver function (Zhao et al., 2022). The gut microbiota and related metabolites play important roles in the treatment of NAFLD, which has become a popular area of research (Hui et al., 2023; Vallianou et al., 2021). For example, the abundances of *Clostridium*, *Anaerobacter*, *Lactobacillus*, taurohyodeoxycholic acid, and taurocholic acid are increased in NAFLD patients (Tilg et al., 2021; Zheng et al., 2024; Wang et al., 2021). However, the effects of XFD on NAFLD, the gut microbiota, and BA-related mechanisms remain unclear. In this study, we observed the effects of XFD on the gut microbiota by analyzing the contents of BAs in the feces, liver, and serum of NAFLD patients via macrogenomics and metabolomics to further assess the mechanism of XFD in the treatment of NAFLD.

## Materials and methods

### Animals and experimental design

Five- to six-week-old male Wistar rats were obtained from Jinan Pengyue Experimental Animal Breeding Co., Ltd. (Jinan, China; certificate no. 370726230100193163). The rats were acclimatized in a specific pathogen-free animal room that was maintained at 22 ± 2°C with 50–70% humidity on a 12-h light/dark cycle and provided water and food.

After acclimatization for 1 week, the eight rats in the control (Con) group were fed standard chow for 8 weeks. In addition, 32 rats were fed a high-fat diet (HFD) for 8 weeks. NAFLD was confirmed via liver pathology in three rats randomly selected from the HFD group. On the basis of their relatively high body weights, 24 rats were randomly divided into three groups (*n* = 8/group). The Wistar rats in the Con group (*n* = 8) and the NAFLD model (Mod) group received distilled water via oral gavage. The OCA group was administered 100 mg/kg/d OCA (Shanghai Yuanye Bio-Technology Co., Ltd., China) via gavage (Xin et al., 2021), and the XFD group was administered 18 g/kg/d XFD (First Affiliated Hospital of Anhui University of Chinese Medicine, China) via gavage. This rat XFD dosage is six times greater than the human dosage (Zhao and Fang, 2024). The appropriate treatment was administered once a day, and the treatment course lasted 12 weeks.

XFD is composed of *Bambusae caulis in Taeniam* (Anhui Puren Traditional Chinese Medicine Pieces Co., Ltd., 10 g), *Atractylodis Rhizoma* (Bozhou Huqiao Pharmaceutical Co., Ltd., 15 g), *Bupleuri Radix* (Anhui Xiehecheng Pharmaceutical Co., Ltd., 10 g), *Aurantii Fructus* (Anhui Puren Traditional Chinese Medicine Pieces Co., Ltd., 15 g), *Scutellariae Radix* (Anhui Xiehecheng Pharmaceutical Co., Ltd., 10 g), *Curcumae Radix* (Anhui Puren Traditional Chinese Medicine Pieces Co., Ltd., 15 g), *Corydalis Rhizoma* (Anhui Boyao Qiancao National Pharmaceutical Co., Ltd., 15 g), *Paeoniae Radix Alba* (Anhui Xiehecheng Pharmaceutical Co., Ltd., 20 g), *Taraxaci Herba* (Anhui Xiehecheng Pharmaceutical Co., Ltd., 20 g), *Crataegi Fructus* (Bozhou Huqiao Pharmaceutical Co., Ltd., 15 g), *Setaria italica* *(L.) Beauv* (Bozhou Huqiao Pharmaceutical Co., Ltd., 20 g), and *Plantaginis Herba* (Bozhou Huqiao Pharmaceutical Co., Ltd., 15 g). XFD was soaked in 1,800 mL of water. The XFD was boiled at high heat and then simmered at low heat for 30 min. The liquid medicine was collected. XFD was subsequently boiled for a second time as previously described. The liquid mixture was mixed and concentrated to 50 mL. The XFD concentration was 3.6 g/mL.

### Examination of baseline indicators

Body weights were measured via a weight scale. After 12 h of fasting, the fasting blood glucose (FBG) level was measured via the tail vein with a Roche ACCU-CHEK Performa glucometer (Basel, Switzerland). The rats were subsequently anesthetized via intraperitoneal injection of 30 mg/kg pentobarbital sodium (Merck, United States). Blood samples were collected from the abdominal aorta and centrifuged at 4,000 revolutions per minute (rpm) for 10 min. Furthermore, the entire liver tissue samples were weighed, and the liver lobe and white fat from the same area were preserved. Feces were removed by dissecting the ileum. The serum, liver, and feces were stored at −80°C.

The liver was weighed, ground, and centrifuged, after which the supernatant was collected. The levels of total cholesterol (TC), triglyceride (TG), alanine aminotransferase (ALT), and aspartate aminotransferase (AST) in the serum and the levels of TG and TC in the liver were determined using a Chemray 800 fully automatic biochemical analyzer (Rayto Life and Analytical Sciences Co., Ltd., China). The fasting insulin (FIns; American Laboratory Products Company, United States) and bile salt hydrolase (BSH; Jiangsu Medical Industrial Co., Ltd., China) concentrations were analyzed via enzyme-linked immunosorbent assay (ELISA) kits according to the manufacturers’ instructions. The homeostasis model assessment–insulin resistance (HOMA–IR) score was calculated as FIns (μIU/mL) × FBG (mmol/L) ÷ 22.5.

### Histological examination

The liver and white fat portions were fixed in a tube containing a 4% paraformaldehyde solution for 24 h. After dehydration, the samples were embedded in paraffin wax, sliced into 4 μm sections, and stained with hematoxylin and eosin (HE).

The liver and white fat tissues were embedded in optimal cutting temperature compound to prepare frozen sections. The samples were then cut and immersed in oil red O staining solution for 8–10 min before removal and subsequent immersion in each of two cylinders of 60% isopropanol for 3 s and 5 s for differentiation. The slices were then soaked in each of 2 tanks of pure water for 10 s, sliced, immersed in hematoxylin counterstain for 3–5 min, and then washed in each of 3 tanks of pure water for 5, 10, and 30 s. The samples were subsequently differentiated for 2–8 s, washed in each of two tanks of distilled water for 10 s, returned to the staining solution for 1 s, and then immersed in each of two tanks of tap water for 5 s and 10 s. Finally, the slices were sealed with a glycerol gelatin sealing agent. Aipathwell (Servicebio) software was used to identify the positive and tissue areas within the samples. The ratio of oil red O-stained area was calculated as the stained area/tissue area. Images were captured under an Eclipse Ci-L microscope (Nikon Corporation, Japan).

### Metagenomic sequencing and analyses

Total genomic DNA was extracted from the fecal samples using an E.Z.N.A.^®^ Soil DNA Kit (Omega Bio-tek, United States). The extracted DNA was randomly fragmented to a size of approximately 400 bp using a Covaris M220 instrument (Gene Company Limited, China). A paired-end library was constructed with NEXTFLEX Rapid DNA-Seq (Bios Scientific, United States). Adapters containing the complements of the sequencing primer hybridization sites were ligated to the blunt ends of the fragments. Paired-end sequencing was performed on an Illumina NovaSeq instrument (Illumina, Inc., United States) at Majorbio Bio-Pharm Technology Co., Ltd. (Shanghai, China) using NovaSeq reagent kits (Illumina, Inc., United States).

Metagenomic data were assembled using MEGAHIT (Li et al., 2015)^1^. The predicted open reading frames with lengths ≥100 bp were retrieved and translated into amino acid sequences via the National Center for Biotechnology Information translation table. High-quality reads were aligned to non-redundant gene catalogs to calculate gene abundance with 95% identity via SOAPaligner (Li et al., 2008)^2^. Representative sequences of the non-redundant gene catalog were aligned to the non-redundant database via Diamond^3^ (Buchfink et al., 2015) for taxonomic annotation. Kyoto Encyclopedia of Genes and Genomes (KEGG) annotation was conducted against the KEGG database via Diamond.

### BA determination and analyses

The 47 BA standards were weighed and prepared as working standard solutions. These standards and the BA isotope standard solutions were used for analysis. The standard solution was added to 100 μL of the sample, which was mixed for 30 s and sonicated at low temperature for 30 min before being stored at −20°C for 30 min. The mixture was subsequently centrifuged at 4°C for 15 min at 13000 rcf, the supernatant was removed, and the solvent was removed by a stream of nitrogen. Then, 100 μL of 50% acetonitrile was added, and the mixture was vortexed for 30 s, sonicated at low temperature for 10 min (5°C, 40 kHz), and centrifuged at 4°C for 15 min at 13,000 rcf, after which the supernatant was removed for liquid chromatography–tandem mass spectrometry (LC–MS/MS) analysis (de Groot et al., 2020; Jung et al., 2021).

LC–MS/MS analysis of the samples was conducted on an ExionLC AD system coupled with a QTRAP^®^ 6,500+ mass spectrometer (Sciex, United States) at Majorbio Bio-Pharm Technology Co., Ltd. (Shanghai, China). In brief, the samples were separated via a Waters BEH C18 column (150 × 2.1 mm, 1.7 μm) at 50°C, and the mass spectrometric data were collected with a SCIEX QTRAP 6500+ mass spectrometer equipped with an electrospray ionization (ESI) source operating in negative ion mode. The raw LC–MS/MS data were imported into SCIEX software OS, and the BA concentrations were calculated according to the standard curve.

### Statistical analysis

SPSS 23.0 (New York, United States) and GraphPad Prism 5.0 (San Diego, United States) were used for statistical analyses and figure generation (Zhao and Fang, 2024). Normality and homogeneity of variance tests were conducted on the data. The experimental results are expressed as the means ± standard deviations. Multiple group comparisons were conducted via one-way analysis of variance (ANOVA) followed by a least significant difference test, Dunnett’s T3 test, or the Kruskal–Wallis test. Principal coordinate analysis (PCoA) was used to display the changes in classification with R software (v3.1.1), mixOmics, and the ade4 package. A Venn diagram was constructed with R software (v3.1.1). The KEGG pathways were predicted via PICRUSt2 v2.2.0-b and R (v3.4.10). ImageGP was used for intergroup pathway analysis. Functional difference analysis was performed via the Wilcoxon test. Receiver operating characteristic (ROC) curves were constructed, the areas under the curve (AUCs) were calculated, decision curve analysis was performed, and correlation network diagrams were generated with R software (v4.2.1). *p*-values <0.05 were considered to indicate statistical significance.

## Results

### Effects of XFD on baseline indicators

Compared to the Con group, the Mod group presented significant increases in body weight and liver weight (*p* < 0.01). Compared to those in the Mod group, the body weights and liver weights in the OCA and XFD groups were significantly lower (*p* < 0.01). In addition, among the four groups, there were no significant differences in the FBG or FIns levels or HOMA–IR scores (*p* > 0.05), as shown in Table 1.

**Table 1:** Baseline characteristics.

Characteristic	Con	Mod	OCA	XFD
Body weight (g)	419.75 ± 42.43	488.25 ± 39.21^a^	424.13 ± 44.06^b^	430.00 ± 41.28^c^
Liver weight (g)	12.60 ± 1.43	15.54 ± 1.77^a^	12.34 ± 0.93^b^	12.44 ± 1.34^d^
FBG (mmol/L)	5.70 ± 0.61	6.09 ± 0.49	6.24 ± 0.59	5.88 ± 0.43
FIns (μIU/mL)	3.34 ± 2.05	3.11 ± 0.56	2.39 ± 0.57	2.30 ± 0.47
HOMA–IR	0.83 ± 0.44	0.84 ± 0.17	0.66 ± 0.18	0.60 ± 0.13

^a^Significant difference between Con and Mod (*p* < 0.01); ^b^significant difference between Mod and OCA (*p* < 0.01); ^c^significant difference between Mod and XFD (*p* < 0.05); ^d^significant difference between Mod and XFD (*p* < 0.01); Con, control; Mod, model; OCA, obeticholic acid; XFD, Xiaohua Funing decoction; FBG, fasting blood glucose; FIns, fasting insulin; HOMA–IR, homeostasis model assessment–insulin resistance.

**Figure 1:**
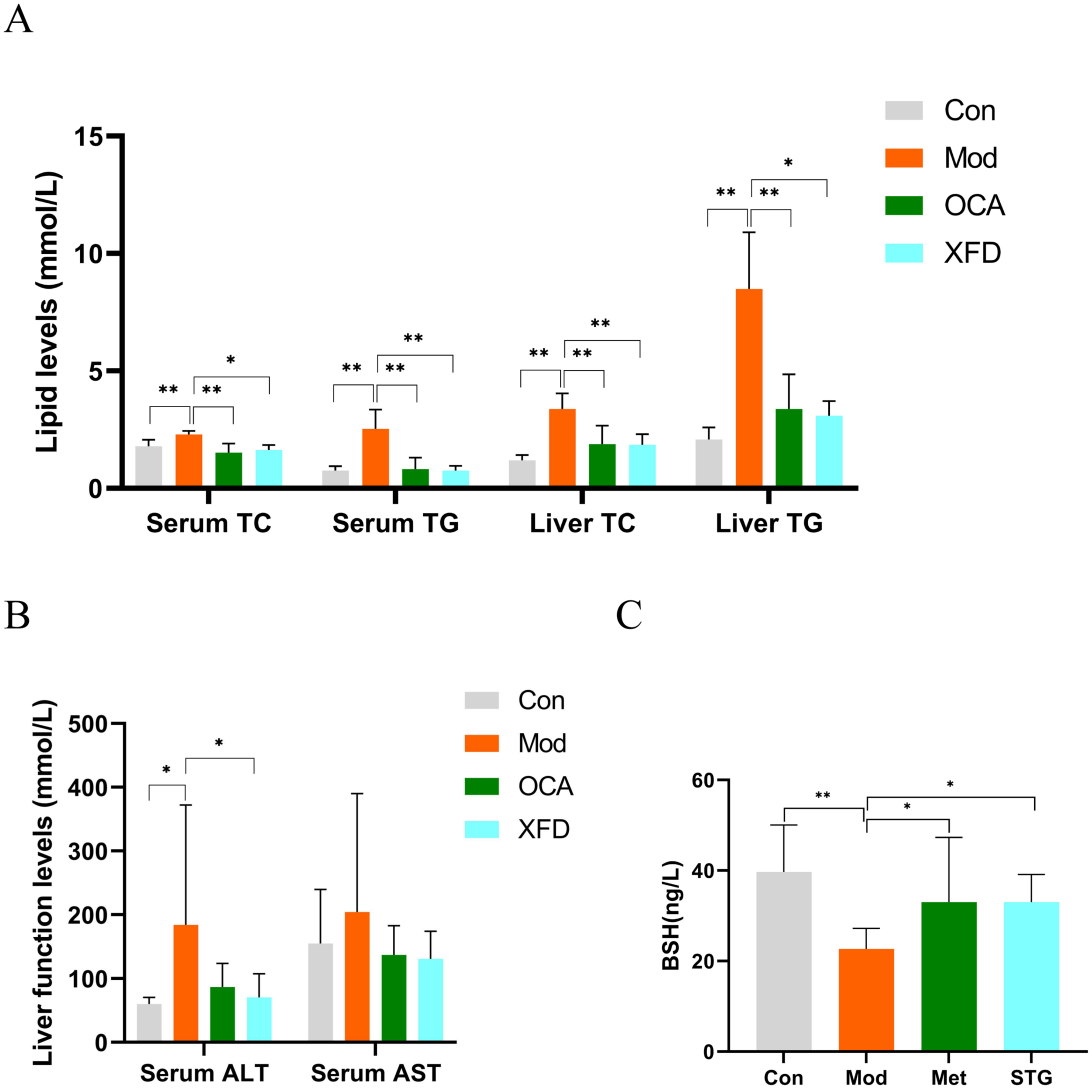
Effects of XFD on baseline indicators. **(A)** Lipid levels. **(B)** Liver function indicators. **(C)** BSH levels. The data are presented as the means ± standard deviations; *n* = 8. Con, control; Mod, model; OCA, obeticholic acid; XFD, Xiaohua Funing decoction; BSH, bile salt hydrolase; TC, total cholesterol; TG, triglyceride; ALT, alanine aminotransferase; AST, aspartate aminotransferase. ^*^p < 0.05, ^**^p < 0.01.

Compared to those in the Con group, the serum and liver TC and TG levels in the Mod group were significantly greater (*p* < 0.01), whereas compared to those in the Mod group, the lipid levels in the OCA and XFD groups were significantly lower (*p* < 0.05, *p* < 0.01) (Figure 1A). Compared to those in the Con group, the serum ALT levels in the Mod group were significantly greater (*p* < 0.05), but compared to those in the Mod group, the serum ALT levels in the XFD group were significantly lower (*p* < 0.05). However, there was no difference in the levels of serum AST among the four groups (Figure 1B). Compared to those in the Con group, the BSH levels in the Mod group were significantly lower (*p* < 0.01), and compared to those in the Mod group, the BSH levels in the OCA and XFD groups were significantly greater (*p* < 0.05) (Figure 1C).

### Effects of XFD on pathological morphology

**Figure 2:**
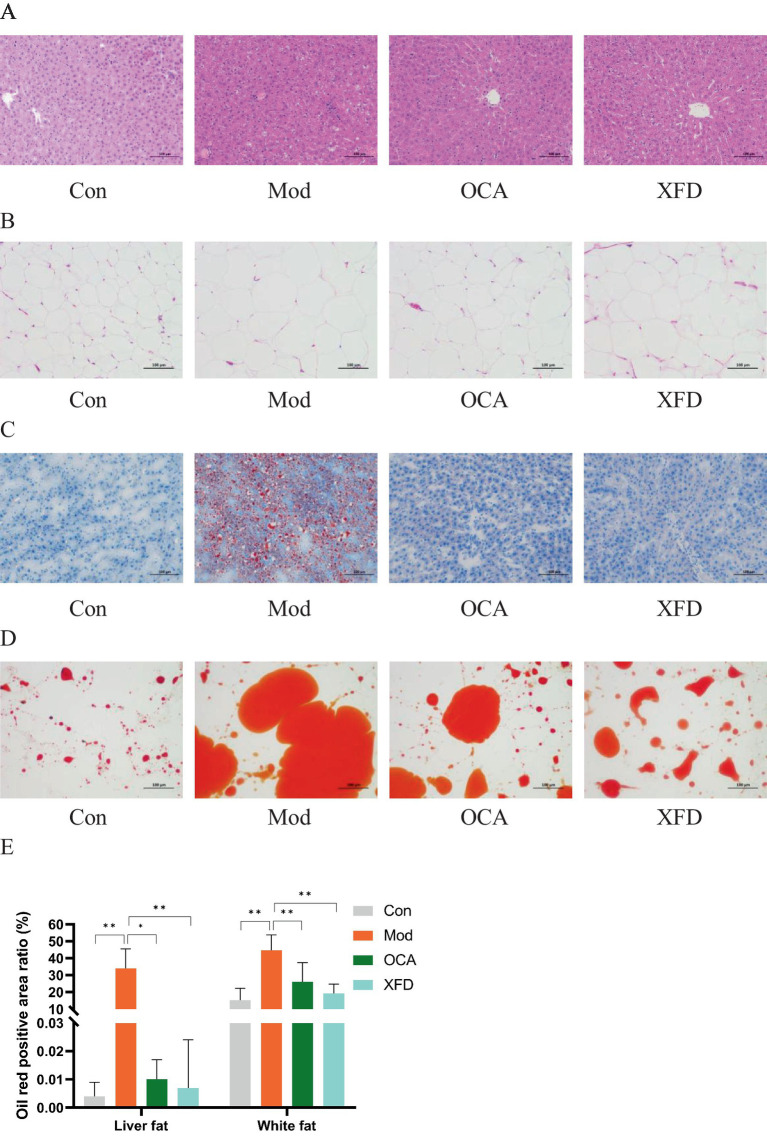
Effects of XFD on pathological morphology. **(A)** HE-stained images of the livers and **(B)** white adipose tissues. **(C)** Oil red O staining images of the livers and **(D)** white adipose tissues. **(E)** Oil red O-positive areas in the adipocytes of the livers and white adipose tissues. The data are presented as the means ± standard deviations; *n* = 3. Con, control; Mod, T2DM model; OCA, obeticholic acid; XFD, Xiaohua Funing decoction. Images are shown at 200× magnification; scale bar, 100 μm. *p < 0.05, **p < 0.01.

Compared to those in the Con group, a greater degree of hepatocyte steatosis and slight lymphocyte infiltration was observed in the livers of the Mod group. However, hepatocyte steatosis was sporadic in the OCA and XFD groups compared to that in the Mod group (Figure 2A). Moreover, the volume of adipocytes in white adipose tissue in the Mod group was greater than that in the Con group, and the OCA and XFD groups had fewer adipocytes than the Mod group (Figure 2B).

In addition, the oil red O-positive areas of adipocytes in the liver and white adipose tissue were significantly greater in the Mod group than those in the Con group (*p* < 0.01), and compared to those of the Mod group, the oil red O-positive areas of the OCA and XFD groups were significantly lower (*p* < 0.05, *p* < 0.01) (Figures 2C–E).

### Effects of XFD on the gut microbiota

**Figure 3:**
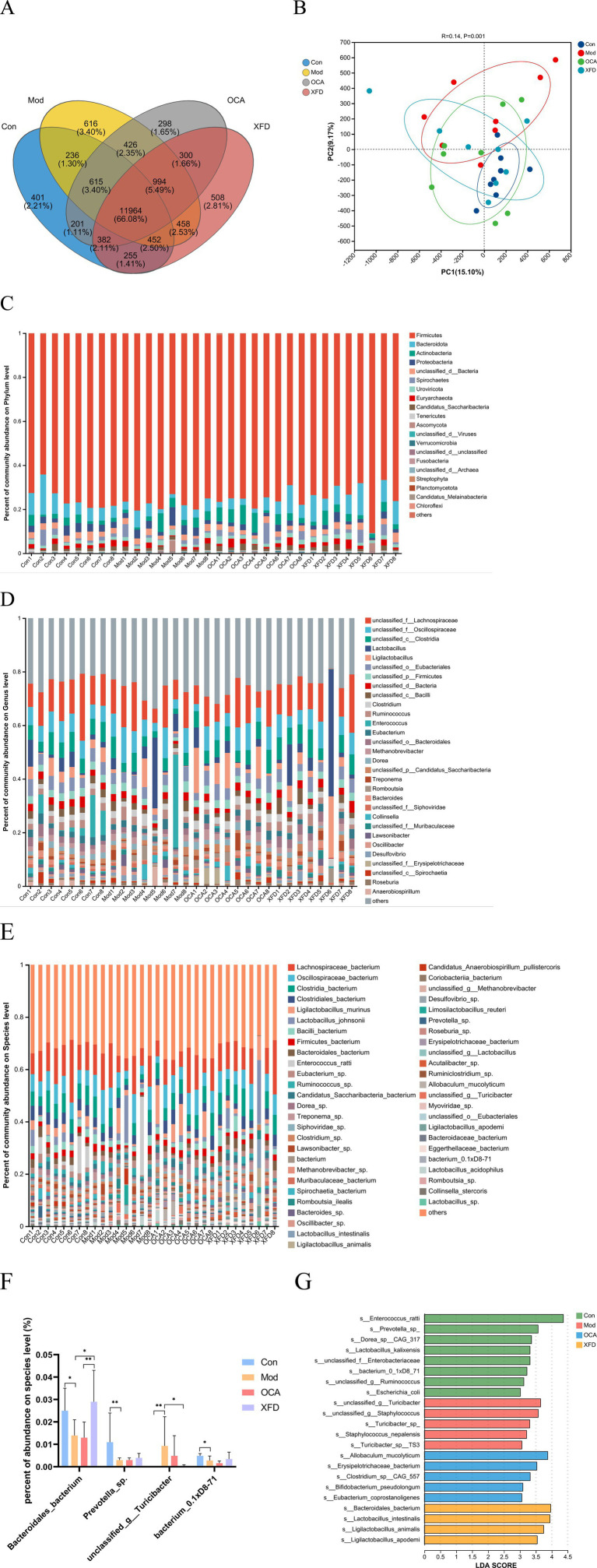
Comparison of the gut microbiota. **(A)** Common and unique species in the gut microbiota. **(B)** Beta diversities of the gut microbiota determined by PCoA. **(C)** Composition of each gut microbiota at the phylum level. **(D)** Comparison of each gut microbiota at the genus level. **(E)** Composition of each gut microbiota at the species level. **(F)** Abundances of each species in the gut microbiota. **(G)** Dominant gut microorganisms at the species level. The data are presented as the means ± standard deviations; *n* = 8. Con, control; Mod, model; OCA, obeticholic acid; XFD, Xiaohua Funing decoction; PCoA, principal coordinate analysis; LDA, linear discriminant analysis. ^*^p < 0.05, ^**^p < 0.01.

A total of 46,372,471 clean reads were obtained for a total of 18,105 species. Notably, 11,964 common species were found among the four groups. A total of 401 unique species were identified in the Con group, 616 in the Mod group, 508 in the XFD group, and 298 in the OCA group (Figure 3A). PCoA revealed differences in the beta diversity of the gut microbiota among the four groups. The Mod group was separated from the Con group, and the OCA and XFD groups were separated from the Mod group (Figure 3B).

The top 20 phyla, 30 genera, and 50 species identified in the gut microbiota are listed in Figures 3C–E. The main microorganisms were not significantly different between the OCA and XFD groups and the Mod group at the phylum and genus levels (*p* > 0.05). Compared to those of the top 20 species in the Con group, the abundances of *Bacteroidales_bacterium*, *Prevotella_*sp., and *bacterium_0.1xD8-71* were significantly lower in the Mod group (*p* < 0.05 or *p* < 0.01), whereas the abundance of *unclassified_g_Turicibacter* was significantly greater (*p* < 0.01). Compared to that in the Mod group, the abundance of *Bacteroidales_bacterium* was significantly greater in the XFD group (*p* < 0.05), and the abundance of *unclassified_g_Turicibacter* was significantly lower (*p* < 0.05) (Figure 3F). On the basis of a linear discriminant analysis (LDA) score > 3.0, 22 species in the four groups were defined as dominant components of the gut microbiota. Notably, *unclassified_g_Turicibacter*, *unclassified_g_Staphylococcus*, *Turicibacter_*sp., *Staphylococcus_nepalensis*, and *Turicibacter_*sp*_TS3* were significantly enriched in the Mod group (*p* < 0.01); *Erysipelotrichaceae_bacterium*, *Allobaculum_mucolyticum*, *Clostridium_*sp*_CAG_557*, *Bifidobacterium_pseudolongum*, and *Eubacterium_coprostanoligenes* were significantly enriched in the OCA group (*p* < 0.05 or *p* < 0.01); and *Ligilactobacillus_animalis*, *Lactobacillus_intestinalis*, *Ligilactobacillus_apodemi*, and *Bacteroidales_bacterium* were significantly enriched in the XFD group (p < 0.05), as shown in Figure 3G. According to LDA and differential analysis, *Bacteroidales_bacterium* may be a crucial species in the XFD group.

### Effects of XFD on the function of the gut microbiota

**Figure 4:**
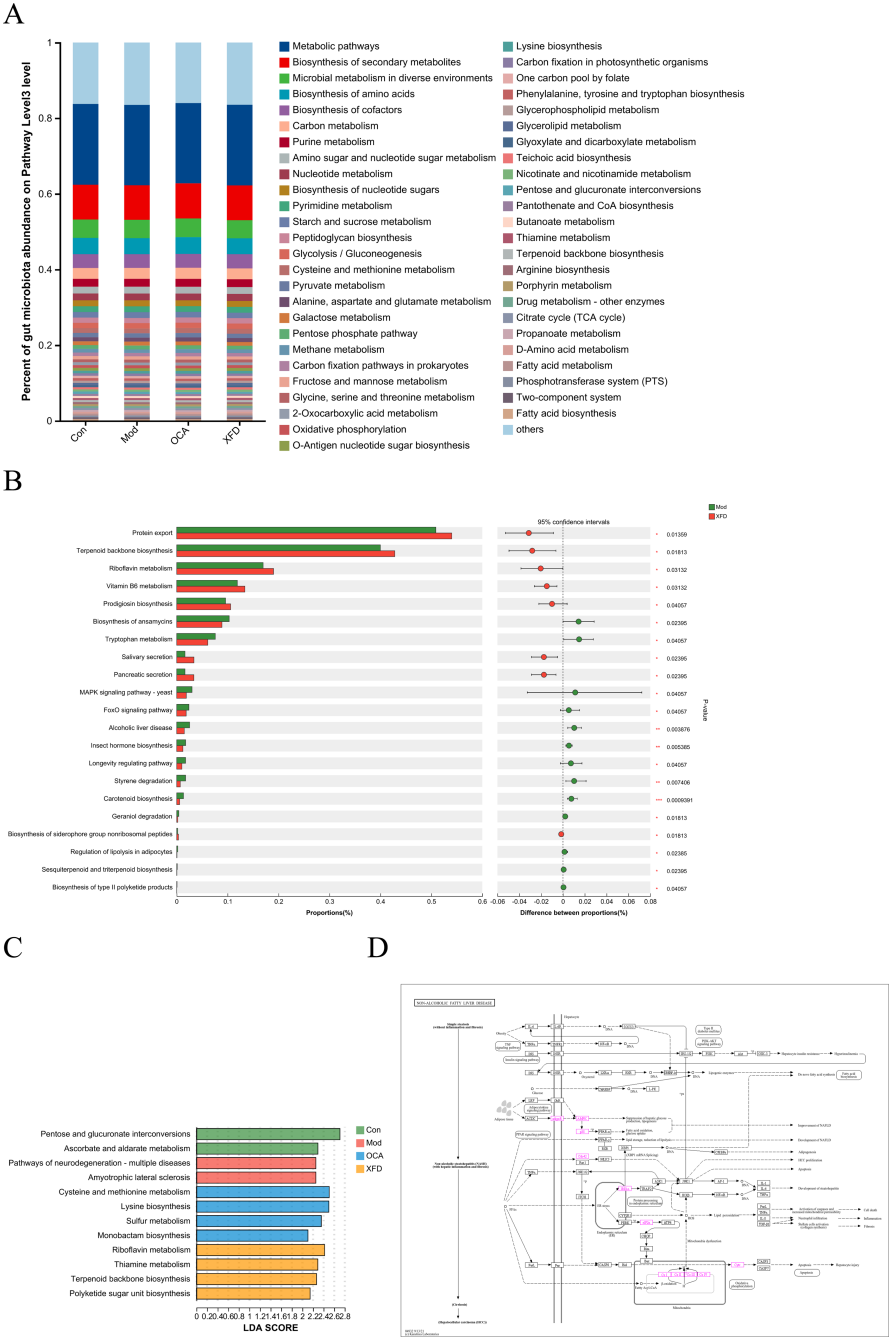
Comparisons of the functions of the gut microbiota. **(A)** KEGG pathway functional annotations of the metabolomic data at the third level. **(B)** Analysis of the enriched KEGG pathways between the Mod and XFD groups. **(C)** The dominant KEGG pathways at the third level. **(D)** Analysis of genes involved in NAFLD-related signaling pathways according to KEGG analysis. *n* = 8. Con, control; Mod, T2DM model; OCA, obeticholic acid; XFD, Xiaohua Funing decoction; NAFLD, non-alcoholic fatty liver disease; KEGG, Kyoto Encyclopedia of Genes and Genomes; LDA, linear discriminant analysis. ^*^p < 0.05, ^**^p < 0.01, ^***^p < 0.001.

A comparison of the predicted gene sequences with those in the KEGG database revealed 12,615 KEGG functional annotations. The KEGG pathway enrichment analysis was performed at the third level, with an abundance of ≥0.0045. The metabolomic analysis revealed that NAFLD was related to the biosynthesis of amino acids; biosynthesis of cofactors; pyrimidine metabolism; glycolysis/gluconeogenesis; pyruvate metabolism; alanine, aspartate, and glutamate metabolism; peptidoglycan biosynthesis; glyoxylate and dicarboxylate metabolism; pantothenate and CoA biosynthesis; starch and sucrose metabolism; cysteine and methionine metabolism; butanoate metabolism; terpenoid backbone biosynthesis; arginine biosynthesis; fatty acid metabolism; glycerolipid metabolism; and thiamine metabolism (Figure 4A).

The XFD treatment affects a series of pathways that attenuate NAFLD in rats; 21 of these pathways that displayed differences in protein expression are shown in Figure 4B. These pathways include sesquiterpenoid and triterpenoid biosynthesis, regulation of lipolysis in adipocytes, vitamin B6 metabolism, riboflavin metabolism, and terpenoid backbone biosynthesis. The KEGG functions of the gut microbiota with an LDA score > 2.0 were defined as dominant, which led to the selection of 12 signaling pathways across the four groups. In the Mod group, pathways related to neurodegeneration and multiple diseases were significantly enriched in differentially expressed proteins (*p* < 0.05), and in the XFD group, pathways related to cysteine and methionine metabolism, lysine biosynthesis, sulfur metabolism, and monobactam biosynthesis were significantly enriched in differentially expressed proteins (*p* < 0.05) (Figure 4C). Moreover, 43 genes related to NAFLD were identified via KEGG analysis. These genes are related to the AMPK signaling pathway, the AMPK/p38 signaling pathway, the cdc42 signaling pathway, and the IRE1α pathway (Figure 4D).

### Effects of XFD on BA levels

**Figure 5:**
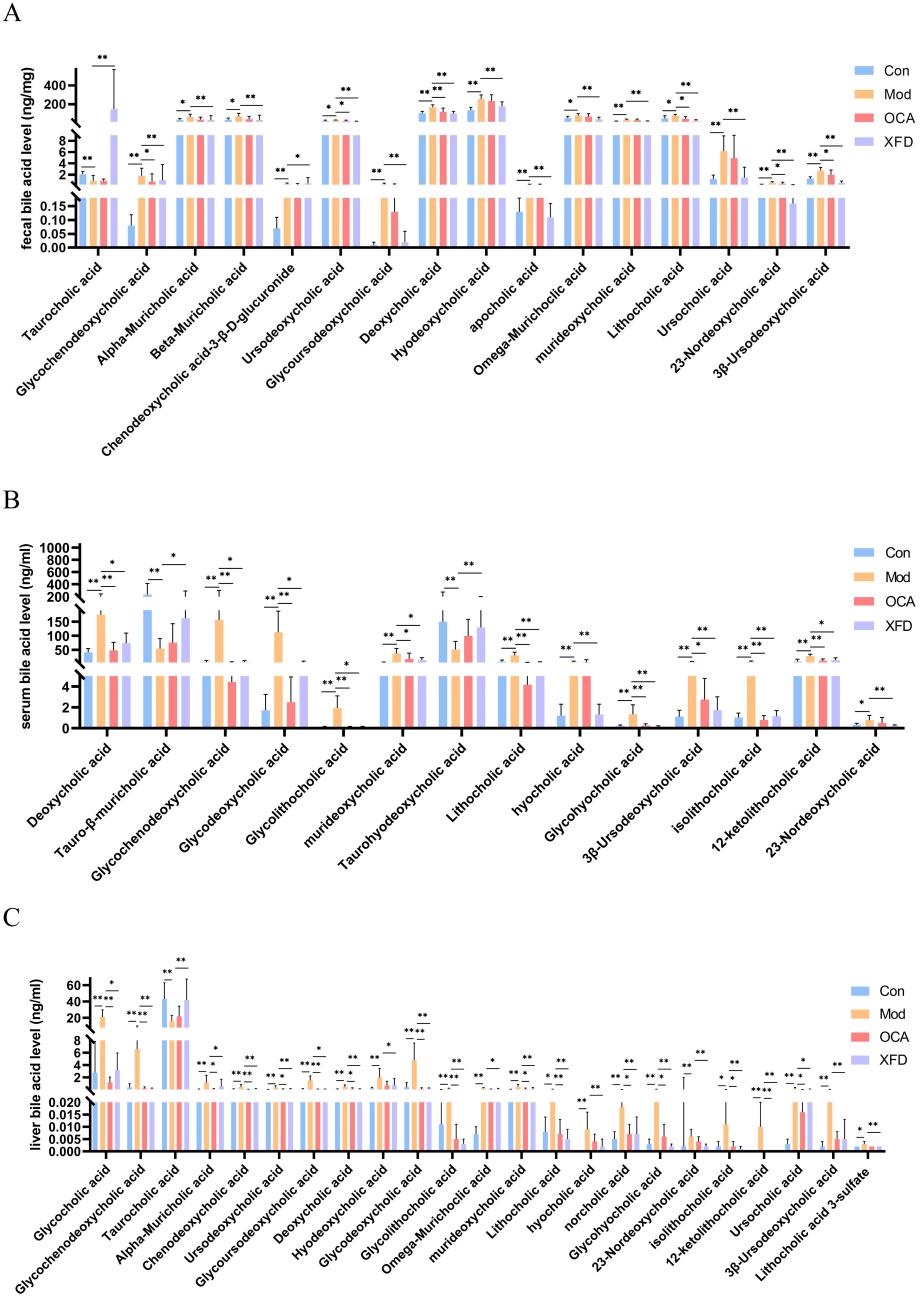
Comparison of BAs. **(A)** Fecal, **(B)** liver, and **(C)** serum contents of BAs regulated by XFD. Con, control; Mod, model; OCA, obeticholic acid; XFD, Xiaohua Funing decoction. *n* = 8. ^*^p < 0.05, ^**^p < 0.01.

Seventeen differentially abundant BAs were detected in the fecal samples (Figure 5A), whereas 24 differentially abundant BAs were detected in the serum and liver samples (Figures 5B,C). On the basis of the above findings, XFD normalized the levels of 16, 23, and 14 BAs in the feces, liver, and serum, respectively. Among 16, 23, and 14 BAs, XFD can normalize the levels of six common differentially abundant BAs: glycochenodeoxycholic acid, deoxycholic acid, murideoxycholic acid, lithocholic acid, 23-nordeoxycholic acid, and 3β-ursodeoxycholic acid.

### Effects of XFD on potential biomarkers

**Figure 6:**
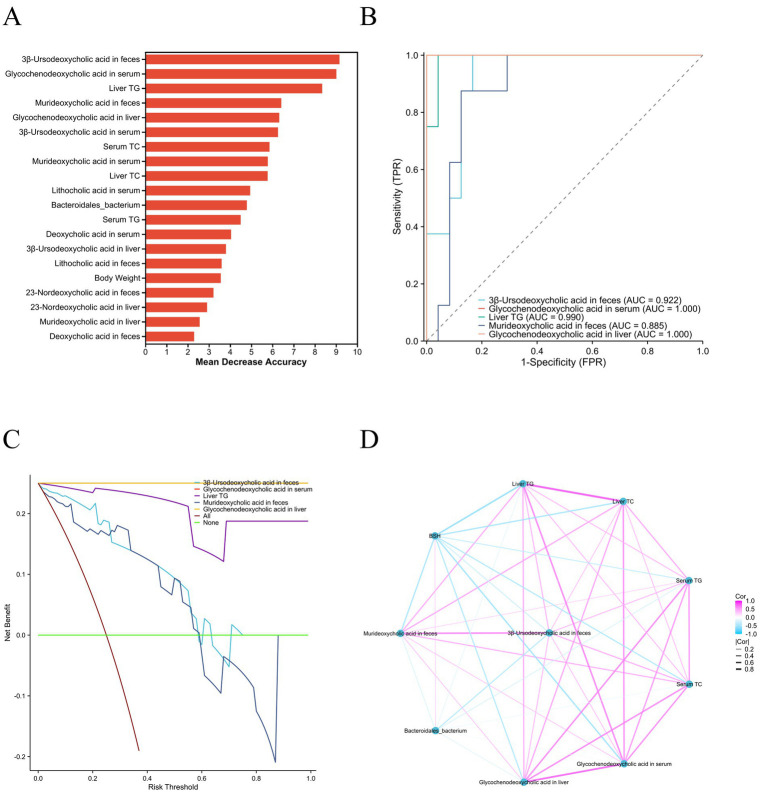
Potential biomarkers of NAFLD. **(A)** Analysis of the importance of potential biomarkers of NAFLD. **(B)** AUC of potential biomarkers. **(C)** Decision curve analyses of the potential biomarkers. **(D)** Correlation analyses of the potential biomarkers. NAFLD, non-alcoholic fatty liver disease; AUC, area under the curve; TG, triglyceride; TC, total cholesterol; TPR, true positive rate; FPR, false positive rate.

Random forest analysis was conducted on the basis of the difference indices of 7 clinical indicators, 18 BAs, BSH, and *Bacteroidales_bacterium* to identify the 5 top biomarkers for the NAFLD diagnostic model. These indicators included 3β-ursodeoxycholic acid in feces, glycochenodeoxycholic acid in the serum, liver TGs, murideoxycholic acid in feces, and glycochenodeoxycholic acid in the liver (Figure 6A). The AUC of glycochenodeoxycholic acid in the serum and liver was 1.0 for NAFLD patients (Figure 6B). Importantly, there are more advantages than disadvantages in the use of glycochenodeoxycholic acid levels in the serum and liver as clinical biomarkers of NAFLD according to the decision curve analysis (Figure 6C). The correlation coefficient between the serum and liver levels of glycochenodeoxycholic acid was 0.799. Furthermore, there were negative correlations between BSH abundance and the levels of glycochenodeoxycholic acid in the serum and liver and between BSH abundance and the level of murideoxycholic acid in the feces (Figure 6D).

## Discussion

Lipid accumulation is a characteristic of NAFLD. Dysbiosis of liver metabolism results from the integration of signals from the gut microbiota and BAs (Albillos et al., 2020). The distinct microbiota profile of NAFLD patients has not been overlooked (Vallianou et al., 2021). The gut microbiota produces or modulates BAs directly in the liver, which in turn activates hepatic FXR signaling and G protein-coupled bile acid receptor signaling to regulate lipid metabolism (Rodríguez-Pastén et al., 2023). Although XFD has long been used clinically in China, the underlying mechanism by which XFD alleviates NAFLD is still unclear. In the Mod group, increased body and liver weights, elevated serum and liver lipid levels, abnormal liver function, and lipid accumulation in the liver and white adipose tissue were observed, all of which are representative manifestations of NAFLD. Given that XFD has good potential in regulating lipid metabolism for NAFLD treatment, we further explored the mechanisms underlying the actions of XFD.

According to TCM reports on NAFLD, the Si Miao formula can alter *Akkermansia muciniphila*. Berberine can restore *Streptococcus* and *Prevotella* (Han et al., 2021; Yue et al., 2019). Our results revealed that the abundance of *Bacteroidales_bacterium* was related to the beneficial effects of XFD on NAFLD as *Bacteroidales_bacterium* has lipid-lowering effects (Yang et al., 2024). In addition, Liu et al. (2023) reported that *Bacteroidales_bacterium* is related to lipid metabolic disorders. We found that the abundance of the key biomarker *Bacteroidales_bacterium* was significantly lower in the Mod and Con groups. Another study revealed that the TG, TC, ALT, and AST levels in serum are significantly negatively correlated with the relative abundance of *Bacteroidales_bacterium* (Zhang et al., 2023). Our research yielded similar results. A characteristic of neurodegeneration in multiple diseases, including NAFLD, is amyloid plaque deposition (Chintapaludi et al., 2020). In our study, this pathway was significantly activated in the Mod group. In addition, the FoxO and MAPK signaling pathways may be involved in NAFLD development. Moreover, FOXO genes inhibit the proliferation and transdifferentiation of hepatic stellate cells, whereas MAPK is involved in oxidative stress and inflammatory reactions (Dong, 2017; Fang et al., 2023). These findings suggest that XFD can ameliorate NAFLD via these two signaling pathways. Tryptophan metabolic disorders have been observed in NAFLD patients (Wu et al., 2024). XFD has a protective effect against NAFLD by normalizing the tryptophan metabolic profile. Vitamin B6 influences fat metabolism, and patients with NAFLD have been found to be deficient in this vitamin (Kobayashi et al., 2021). Notably, XFD can increase vitamin B6 levels to alleviate NAFLD. Depleting flavin adenine dinucleotide pools by consuming a riboflavin metabolite-deficient diet has been shown to cause phenotypes similar to NAFLD (Masschelin et al., 2023), but XFD intervention normalizes riboflavin metabolism. Terpenoid backbones undergo a series of enzymatic redox modifications to ultimately produce various terpenoid natural products (Rymut et al., 2022). Furthermore, the genes in the terpenoid backbone biosynthesis pathway were expressed at lower levels in the Mod group than those in the XFD group. These findings indicate that XFD can increase the activity of this pathway. TCM is effective in delaying the progression of NAFLD through its effects on the gut microbiota and BAs (Sun et al., 2022). The Huanglian–Hongqu herb pair primarily modulates total BAs (Zhang et al., 2024). Astragalus polysaccharides can alter taurohyodeoxycholic acid to protect against NAFLD (Zheng et al., 2024). *Bacteroidales* is involved in BA metabolism (Zafar and Saier, 2021). BSH is an amino N-acyltransferase that regulates BA metabolism (Rimal et al., 2024). Thus, high BSH activity can lead to the hydrolysis of BA conjugates, trigger the consumption of liver cholesterol, and reduce hyperlipidemia in the body (Zhang et al., 2021). The regulation of BA metabolism by the gut microbiota is dependent on the effects of BSH on NAFLD development (Li et al., 2023). The results of this study suggest that consuming an HFD reduces BSH levels, which may lead to the development of NAFLD and is related to a change in the abundance of *Bacteroidales_bacterium*. BSH concentrations in the Mod group were negatively correlated with murideoxycholic acid abundance in the feces and glycochenodeoxycholic acid abundance in the serum and liver. Targeted liver, feces, and serum metabolomic analyses revealed that six BAs, namely, glycochenodeoxycholic acid, deoxycholic acid, murideoxycholic acid, lithocholic acid, 23-nordeoxycholic acid, and 3β-ursodeoxycholic acid, were negatively correlated with *Bacteroidales_bacterium* abundance and were present at relatively low concentrations in the XFD group. Glycochenodeoxycholic acid levels were significantly greater in female patients or chickens with NAFLD (Fitzinger et al., 2024; Yang et al., 2024) and are a potential biomarker for the diagnosis of NAFLD (Huang et al., 2024). Glycochenodeoxycholic acid can induce hepatocyte death or NAFLD by inhibiting endoplasmic reticulum stress (Wu et al., 2022). However, metformin could be used to treat NAFLD by protecting against glycochenodeoxycholic acid-induced hepatocyte death (Woudenberg-Vrenken et al., 2013). The levels of glycochenodeoxycholic acid in the serum and liver are highly correlated. Glycochenodeoxycholic acid has the greatest net benefit for diagnosing NAFLD according to the AUC and decision curve analysis. XFD may serve as a therapeutic modality for NAFLD by reducing glycochenodeoxycholic acid levels. These effects may inhibit endoplasmic reticulum stress and decrease the phosphorylation of the JNK signaling pathway (Wu et al., 2022).

Many obese individuals have NAFLD, and obesity increases the level of deoxycholic acid (Yoshimoto et al., 2013), which is known to cause DNA damage. Hydrogen sulfide can decrease deoxycholic acid expression, thereby modulating the expression of target genes related to lipid metabolism to relieve NAFLD (Xu et al., 2022). This phenomenon was also observed in the XFD group. This finding may be related to the regulation of the liver’s FXR-SHP or TGR5 signaling pathway (Shi et al., 2021; Gillard et al., 2021). Murideoxycholic acid influences cholesterol metabolism and BA metabolism (Cohen et al., 1991). Sini decoction modulates the NF-κB signaling pathway by regulating murideoxycholic acid (Zhou et al., 2017). In our research, XFD alleviated NAFLD by regulating murideoxycholic acid levels. Lithocholic acid plays a role in adipose tissue browning (Chiang and Ferrell, 2020) and regulates the excretion, absorption, and transport of fats, whereas diosgenin alleviates abnormal lipid metabolism in NAFLD by modulating BAs (Zhou et al., 2022). This finding may also explain why XFD alleviates NAFLD by influencing lithocholic acid. Few reports have noted the protective effects of XFD on NAFLD mediated by the regulation of 3β-ursodeoxycholic acid, as observed in our research. 3β-Ursodeoxycholic acid can be isomerized by gut and liver enzymes to produce ursodeoxycholic acid. However, ursodeoxycholic acid has been shown to have both negative and positive effects on NAFLD in clinical studies (Mahjoubin-Tehran et al., 2021). Compared to healthy controls, individuals with depressive disorder have elevated 23-nordeoxycholic acid levels (Sun et al., 2022). Intriguingly, XFD can decrease 23-nordeoxycholic acid expression. However, the mechanism of this action is unclear. The use of gut microbiota and targeted metabolomics to identify the molecular targets of TCM is novel. These data provide methodological references for future research. This study also has certain limitations as we did not assess downstream indicators of BAs. In the next step, we focus on the impact of XFD on the mRNA and protein expression related to BAs. These results are consistent with the findings from other studies showing that TCM affects the gut microbiota and BAs. However, the specific types and levels are not entirely consistent.

## Conclusion

The effect of NAFLD on lipid metabolism may be mediated through BAs produced by the gut microbiota. In summary, the beneficial effects of XFD on liver lipid accumulation in HFD-fed mice are partly mediated by regulating the abundance of the gut microbiota and the composition and concentration of BAs. XFD normalized the structure of the gut microbiota, especially the abundance of *Bacteroidales_bacterium*. *Bacteroidales_bacterium* belongs to the phylum Bacteroidetes and can affect BAs. XFD also affects the levels of glycochenodeoxycholic acid, murideoxycholic acid, and 3β-ursodeoxycholic acid in the liver, feces, and serum. Therefore, XFD could be a potential complementary or alternative treatment for NAFLD by regulating the gut microbiota and BAs via a novel hypolipidemic pathway.

## Data Availability

The datasets presented in this study can be found in online repositories. The names of the repository/repositories and accession number(s) can be found in the article/supplementary material.

## Footnotes

^1^ https://github.com/voutcn/megahit

^2^ https://github.com/ShujiaHuang/SOAPaligner

^3^ https://github.com/bbuchfink/diamond
